# Supplementary material for: The Systems Biology Research Tool: evolvable open-source software
Source: BMC Syst Biol. 2008 Jun 29;2:55. doi: 10.1186/1752-0509-2-55 (PMC2446383; doi:10.1186/1752-0509-2-55)
Supplement: Additional file 1 — SBRT Archive. An archive of the current version of the Systems Biology Research Tool. [file 1752-0509-2-55-S1.zip › sbrt-1.4.0/doc/developers_guide/api/sbrt/kernel/fba/comp/FbaOptComputer.html]

FbaOptComputer


|  |  |  |
| --- | --- | --- |
| **Overview** \| **Package** \| **Class** \| **Use** \| **Tree** \| **Deprecated** \| **Index** \| **Help** | |  |
| **PREV CLASS**   **NEXT CLASS** | **FRAMES**    **NO FRAMES**     **All Classes** |  |
| SUMMARY: NESTED \| FIELD \| CONSTR \| METHOD | DETAIL: FIELD \| CONSTR \| METHOD |  |


---


## sbrt.kernel.fba.comp Class FbaOptComputer

```
java.lang.Object
  sbrt.kernel.fba.comp.FbaOptComputer
```

**All Implemented Interfaces:**: FluxCvComputer

---

``` public final class FbaOptComputer extends java.lang.Object implements FluxCvComputer ```

This class is the workhorse of all Flux Balance Analysis optimizations.
It provides an interface for communication between FBA experiments
and program solvers.

**Author:**
:   This class was written and documented by Jeremiah Wright
    while in the Wagner lab.

---

| **Nested Class Summary** | |
| --- | --- |
| `class` | `FbaOptComputer.Results`             This class is used to store the results of an FBA optimization problem. |


| **Field Summary** | |
| --- | --- |
| `static double` | `DEFAULT_CONST_TOL`             The default constraint tolerance. |
| `static int` | `DEFAULT_SAFETY_LEVEL`             The default saftey level. |


**Constructor Summary**

`FbaOptComputer(IrrevRxnFluxome fluxome,
ProgramSolver<java.lang.Object,java.lang.Object> solver)`
  
          Constructs a new FBA optimization computer from the
provided fluxome and program solver.

`FbaOptComputer(IrrevRxnFluxome fluxome,
ProgramSolver<java.lang.Object,java.lang.Object> solver,
double constraintTolerance)`
  
          Constructs a new FBA optimization computer from the
provided fluxome, program solver, and constraint tolerance.


| **Method Summary** | |
| --- | --- |
| `protected  void` | `checkChemSpecies(java.lang.String string)`             Ensures the provided string is the name of a chemical species in the fluxome used by this optimization computer. |
| `protected  void` | `checkColExpr(MathExpr<?> e)`             Ensures all the variables in the provided expression are valid column variables. |
| `protected  void` | `checkColumnValue(java.lang.Object columnVariable, double value)`             Ensures the provided numerical value does not violate the defined constraint of the provided column variable. |
| `protected  void` | `checkColumnValue(java.lang.Object columnVariable, java.lang.Double value)`             Ensures the provided numerical value does not violate the defined constraint of the provided column variable. |
| `protected  void` | `checkColumnVariable(java.lang.Object obj)`             Ensures the provided object is column variable. |
| `protected  void` | `checkColumnVariables(java.util.Collection<?> c)`             Ensures each element of the provided collection is column variable. |
| `protected  void` | `checkFluxConstraint(Interval c, java.lang.String rxnName, double tolerance)`             Ensures the provided constraint is a valid flux constraint in the fluxome used by this optimization computer. |
| `protected  void` | `checkNumber(double number)`             Ensures the provided number is finite and not NaN. |
| `protected  void` | `checkRowVariable(java.lang.Object obj)`             Ensures the provided object is row variable. |
| `protected  void` | `checkRxnExpr(MathExpr<java.lang.String> e)`             Ensures all the variables in the provided expression are reaction names. |
| `protected  void` | `checkRxnExprConstraint(Interval c)`             Ensures the provided constraint is valid for reaction name-expressions for the fluxome used by this optimization computer. |
| `protected  void` | `checkRxnName(java.lang.String string)`             Ensures the provided string is the name of a reaction in the fluxome used by this optimization computer. |
| `protected  void` | `checkRxnNames(java.util.Collection<java.lang.String> c)`             Ensures each element of the provided collection is reaction name. |
| `protected  void` | `checkSolutionVector(java.util.Map<?,java.lang.Double> solutionVector)`             Ensures the provided solution vector does not violate the defined constraints of the system. |
| `Interval` | `getColExprConstraint(MathExpr<?> columnVariableExpr)`             Returns the current constraint on the provided expression. |
| `java.util.Map<MathExpr<?>,Interval>` | `getColExprConstraints()`             Returns all current constraints on mathematical expressions of column variables. |
| `java.util.Set<MathExpr<?>>` | `getColExprs()`             Returns the set of all mathematical expressions of column variables. |
| `Interval` | `getColumnConstraint(java.lang.Object columnVariable)`             Returns the current constraint on the provided column variable. |
| `java.util.Map<?,Interval>` | `getColumnConstraints()`             Returns the current constraints for each column variable in the program matrix. |
| `double` | `getColumnMax(java.lang.Object columnVariable)`             Computes the maximum value of the provided column variable. |
| `<T> java.util.Map<T,java.lang.Double>` | `getColumnMaxima(java.util.Collection<T> columnVariables)`             Computes the maximum values of each provided column variable. |
| `double` | `getColumnMin(java.lang.Object columnVariable)`             Computes the minimum value of the provided column variable. |
| `<T> java.util.Map<T,java.lang.Double>` | `getColumnMinima(java.util.Collection<T> columnVariables)`             Computes the minimum values of each provided column variable. |
| `java.util.Set<?>` | `getColumnVariables()`             Returns the set of all column variables in the program matrix. |
| `protected <V> LinearComb<V>` | `getExpr(java.util.Collection<V> columnVariables)`             Converts a collection of column variables to a linear combination. |
| `protected <V> LinearComb<V>` | `getExpr(V columnVariable)`             Converts a column variable to a single-term linear combination. |
| `Interval` | `getFluxConstraint(java.lang.String reactionName)`             Returns the current flux constraint on the reaction with the provided name. |
| `java.util.Map<java.lang.String,Interval>` | `getFluxConstraints()`             Returns all current flux constraints for each reaction in the fluxome used by this optimization computer. |
| `double` | `getFluxMax(java.lang.String reactionName)`             Computes the maximum flux of the reaction with the provided name. |
| `java.util.Map<java.lang.String,java.lang.Double>` | `getFluxMaxima(java.util.Collection<java.lang.String> reactionNames)`             Computes the maximum flux of each reaction with the provided names. |
| `double` | `getFluxMin(java.lang.String reactionName)`             Computes the minimum flux of the reaction with the provided name. |
| `java.util.Map<java.lang.String,java.lang.Double>` | `getFluxMinima(java.util.Collection<java.lang.String> reactionNames)`             Computes the minimum flux of each reaction with the provided names. |
| `IrrevRxnFluxome` | `getFluxome()`             Returns the fluxome used by this optimization computer. |
| `FbaOptComputer.Results` | `getMax(MathExpr<?> objective)`             Returns the results of computing the maximum value of the provided objective function. |
| `<V,E extends MathExpr<V>> java.util.Map<E,java.lang.Double>` | `getMaxima(java.util.Collection<E> objectives)`             Computes the maximum value of each provided objective function. |
| `double` | `getMaximum(MathExpr<?> objective)`             Computes the maximum value of the provided objective function. |
| `FbaOptComputer.Results` | `getMin(MathExpr<?> objective)`             Returns the results of computing the minimum value of the provided objective function. |
| `<V,E extends MathExpr<V>> java.util.Map<E,java.lang.Double>` | `getMinima(java.util.Collection<E> objectives)`             Computes the minimum value of each provided objective function. |
| `double` | `getMinimum(MathExpr<?> objective)`             Computes the minimum value of the provided objective function. |
| `FbaOptComputer.Results` | `getOptimal(MathExpr<?> objective, OptimizationSense sense)`             Returns the results of computing the optimal value of the provided objective function. |
| `FbaOptComputer.Results` | `getOptimal(java.lang.String reactionName, OptimizationSense sense)`             Returns the results of computing the optimal flux of the reaction with the provided name. |
| `java.lang.Class` | `getProgramSolverType()`             Returns the `Class` of the program solver used by this optimization computer. |
| `Interval` | `getRowConstraint(java.lang.Object rowVariable)`             Returns the current constraint on the provided row variable. |
| `java.util.Map<?,Interval>` | `getRowConstraints()`             Returns all the current constraints on row variables. |
| `java.util.Set<?>` | `getRowVariables()`             Returns the set of all row variables in the program matrix. |
| `protected  LinearComb<java.lang.String>` | `getRxnExpr(java.util.Collection<java.lang.String> rxnNames)`             Converts a collection of reaction names to a linear combination. |
| `protected  LinearComb<java.lang.String>` | `getRxnExpr(java.lang.String reactionName)`             Converts a reaction name to a single-term linear combination. |
| `Interval` | `getRxnExprConstraint(MathExpr<java.lang.String> rxnNameExpr)`             Returns the current constraint on the provided expression. |
| `java.util.Map<MathExpr<java.lang.String>,Interval>` | `getRxnExprConstraints()`             Returns all current constraints on mathematical expressions of reaction names. |
| `java.util.Set<MathExpr<java.lang.String>>` | `getRxnExprs()`             Returns the set of all mathematical expressions of reaction names. |
| `double` | `getTolerance()`             Returns the allowable amount by which column variables may violate their defined constraints. |
| `void` | `restoreColExprConstraint(MathExpr<?> columnVarExpr)`             Restores the constraint on the provided expression of column variables to the value contained in the fluxome. |
| `void` | `restoreColExprConstraints()`             Restores the constraints on all mathematical expressions of column variables. |
| `void` | `restoreColExprConstraints(java.util.Collection<? extends MathExpr<?>> colVarExprs)`             Restores the constraints for the provided column variable expressions to those in the provided fluxome. |
| `void` | `restoreColumnConstraint(java.lang.Object columnVariable)`             Restores the constraint for the provided column variable to the value contained in the fluxome. |
| `void` | `restoreColumnConstraints()`             Restores the constraints on all column variables to those contained in the fluxome used by this optimization computer. |
| `void` | `restoreColumnConstraints(java.util.Collection<?> columnVariables)`             Restores the constraints for the provided column variables to those in the provided fluxome. |
| `void` | `restoreFluxConstraint(java.lang.String reactionName)`             Restores the flux constraint for the reaction with the provided name to the value contained in the fluxome. |
| `void` | `restoreFluxConstraints()`             Restores the flux constraints for all reactions to those contained in the fluxome used by this optimization computer. |
| `void` | `restoreFluxConstraints(java.util.Collection<java.lang.String> reactionNames)`             Restores the constraints for the provided reaction names to those in the provided fluxome. |
| `void` | `restoreMatrixElements()`             Restores the program matrix to the stoichiometry matrix of the fluxome used by this computer. |
| `void` | `restoreRowConstraint(java.lang.Object rowVariable)`             Restores the constraint on the provided row variable. |
| `void` | `restoreRowConstraints()`             Restores the constraints on all row variables to those contained in the fluxome used by this optimization computer. |
| `void` | `restoreRowConstraints(java.util.Collection<?> rowVariables)`             Restores the constraints for the provided row variables to those in the provided fluxome. |
| `void` | `restoreRxnExprConstraint(MathExpr<java.lang.String> rxnNameExpr)`             Restores the constraint on the provided flux expression to the value contained in the fluxome. |
| `void` | `restoreRxnExprConstraints()`             Restores the constraints on all mathematical expressions of reaction names. |
| `void` | `restoreRxnExprConstraints(java.util.Collection<? extends MathExpr<java.lang.String>> rxnNameExprs)`             Restores the constraints for the provided reaction name expressions to those in the provided fluxome. |
| `void` | `setColExprConstraint(MathExpr<?> expr, Interval c)`             Changes the constraint on the provided mathematical expression of column variables. |
| `void` | `setColExprConstraints(java.util.Map<? extends MathExpr<?>,Interval> exprConstraints)`             Changes the constraints on more than one mathematical expression of column variables. |
| `void` | `setColumnConstraint(java.lang.Object columnVar, Interval c)`             Changes the constraint of the provided column variable. |
| `void` | `setColumnConstraints(java.util.Map<?,Interval> columnConstraints)`             Changes the constraints on more than one column variable. |
| `void` | `setFluxConstraint(java.lang.String reactionName, Interval c)`             Changes the flux constraint of the reaction with the provided name. |
| `void` | `setFluxConstraints(java.util.Map<java.lang.String,Interval> fluxConstraints)`             Changes the flux constraints on more than one reaction. |
| `void` | `setMatrixElement(java.lang.Object rowVar, java.lang.Object columnVar, double value)`             Sets the specified element of the program matrix to the one provided. |
| `void` | `setMatrixElements(DoubleMatrix<?,?> elements)`             Sets the specified elements of the program matrix to those provided. |
| `void` | `setRowConstraint(java.lang.Object rowVariable, Interval c)`             Changes the constraint of the provided row variable. |
| `void` | `setRowConstraints(java.util.Map<?,Interval> rowConstraints)`             Changes the constraints on more than one row variable. |
| `void` | `setRxnExprConstraint(MathExpr<java.lang.String> expr, Interval c)`             Changes the constraint on the provided mathematical expression of fluxes. |
| `void` | `setRxnExprConstraints(java.util.Map<? extends MathExpr<java.lang.String>,Interval> exprConstraints)`             Changes the constraints on more than one mathematical expression of fluxes. |
| `void` | `setSafetyLevel(int level)`             Sets the safety level. |
| `void` | `setTolerance(double constraintTolerance)`             Sets the constraint tolerance. |

**Methods inherited from class java.lang.Object**

`clone, equals, finalize, getClass, hashCode, notify, notifyAll, toString, wait, wait, wait`

**Field Detail**

### DEFAULT\_SAFETY\_LEVEL

```
public static final int DEFAULT_SAFETY_LEVEL
```

:   The default saftey level.

    **See Also:**: Constant Field Values

---


### DEFAULT\_CONST\_TOL

```
public static final double DEFAULT_CONST_TOL
```

:   The default constraint tolerance.

    **See Also:**: Constant Field Values


**Constructor Detail**

### FbaOptComputer

```
public FbaOptComputer(IrrevRxnFluxome fluxome,
                      ProgramSolver<java.lang.Object,java.lang.Object> solver)
```

:   Constructs a new FBA optimization computer from the
    provided fluxome and program solver.

    **Parameters:**: `fluxome` - the fluxome for which all values will be computed.: `solver` - the program solver used to compute all values.

---


### FbaOptComputer

```
public FbaOptComputer(IrrevRxnFluxome fluxome,
                      ProgramSolver<java.lang.Object,java.lang.Object> solver,
                      double constraintTolerance)
```

:   Constructs a new FBA optimization computer from the
    provided fluxome, program solver, and constraint tolerance.

    **Parameters:**: `fluxome` - the fluxome for which all values will be computed.: `solver` - the program solver used to compute all values.: `constraintTolerance` - the allowed amount by which computed values can violate their defined constraints.


**Method Detail**

### setSafetyLevel

```
public void setSafetyLevel(int level)
```

:   Sets the safety level. Safety levels are used to ensure
    that program solvers do not violate the defined constraints
    of the system. A
    safety level of 0 means no error checking will be done. A
    safety level of 1 means the solution vector will be checked
    for column constraint violations after each optimization.

    :   **Parameters:**: `level` - the saftey level. **Throws:**: `java.lang.IllegalArgumentException` - if the provided value does not equal 0 or 1. **See Also:**: `DEFAULT_SAFETY_LEVEL`, `setTolerance(double)`

---


### setTolerance

```
public void setTolerance(double constraintTolerance)
```

:   Sets the constraint tolerance.

    :   **Parameters:**: `constraintTolerance` - the amount by which computed values can violate their defined constraints. **Throws:**: `java.lang.IllegalArgumentException` - if the provided value is NaN, infinite, or less than 0. **See Also:**: `setSafetyLevel(int)`

---


### getTolerance

```
public double getTolerance()
```

:   Returns the allowable amount by which column variables
    may violate their defined constraints.

    :   **Returns:**: the allowable amount by which column variables may violate their defined constraints.

---


### getProgramSolverType

```
public java.lang.Class getProgramSolverType()
```

:   Returns the `Class` of the program solver used by this
    optimization computer.

    :   **Returns:**: the `Class` of the program solver used by this optimization computer.

---


### getFluxome

```
public IrrevRxnFluxome getFluxome()
```

:   Returns the fluxome used by this optimization computer.

    :   **Specified by:**: `getFluxome` in interface `FluxCvComputer`
    :   **Returns:**: the fluxome used by this optimization computer.

---


### setMatrixElement

```
public final void setMatrixElement(java.lang.Object rowVar,
                                   java.lang.Object columnVar,
                                   double value)
```

:   Sets the specified element of the program matrix to the one provided.
    If either variable does not yet exist in this computer, its
    constraint is set to (-Infinity, Infinity).

    :   **Parameters:**: `rowVar` - the row variable.: `columnVar` - the column variable.: `value` - the new value. **Throws:**: `java.lang.IllegalArgumentException` - if `value` is infinite or NaN.: `java.lang.NullPointerException` - if either variable is `null`.

---


### setMatrixElements

```
public final void setMatrixElements(DoubleMatrix<?,?> elements)
```

:   Sets the specified elements of the program matrix to those provided.
    If any provided variable does not yet exist in this computer, its
    constraint is set to (-Infinity, Infinity).

    :   **Parameters:**: `elements` - a matrix containing the elements to be set. **Throws:**: `java.lang.IllegalArgumentException` - if `value` is infinite or NaN.: `java.lang.NullPointerException` - if either variable is `null`.

---


### restoreMatrixElements

```
public final void restoreMatrixElements()
```

:   Restores the program matrix to the stoichiometry matrix of the fluxome
    used by this computer.

---


### setColumnConstraint

```
public final void setColumnConstraint(java.lang.Object columnVar,
                                      Interval c)
```

:   Changes the constraint of the provided column variable.

    :   **Parameters:**: `columnVar` - the column variable in the program matrix whose constraint is to be changed.: `c` - the new constraint. **Throws:**: `java.lang.IllegalArgumentException` - if `columnVar` is not a column variable in the program matrix.: `java.lang.NullPointerException` - if the provided constraint is `null`. **See Also:**: `setFluxConstraint(String, Interval)`

---


### setFluxConstraint

```
public final void setFluxConstraint(java.lang.String reactionName,
                                    Interval c)
```

:   Changes the flux constraint of the reaction with the provided name.

    :   **Specified by:**: `setFluxConstraint` in interface `FluxCvComputer`
    :   **Parameters:**: `reactionName` - the name of the reaction whose flux constraint is to be changed.: `c` - the new constraint. **Throws:**: `java.lang.IllegalArgumentException` - if `reactionName` is not the name of a reaction in the fluxome used by this computer.: `java.lang.NullPointerException` - if the provided constraint is `null`. **See Also:**: `setColumnConstraint(Object, Interval)`

---


### setRxnExprConstraint

```
public final void setRxnExprConstraint(MathExpr<java.lang.String> expr,
                                       Interval c)
```

:   Changes the constraint on the provided mathematical expression
    of fluxes.

    :   **Specified by:**: `setRxnExprConstraint` in interface `FluxCvComputer`
    :   **Parameters:**: `expr` - a matheamtical expression of reaction names.: `c` - the new constraint. **Throws:**: `java.lang.IllegalArgumentException` - if the variables in the provided expressions are not the names of reactions in the fluxome used by this computer.: `java.lang.NullPointerException` - if either argument is `null`.

---


### setColExprConstraint

```
public final void setColExprConstraint(MathExpr<?> expr,
                                       Interval c)
```

:   Changes the constraint on the provided mathematical expression
    of column variables.

    :   **Parameters:**: `expr` - a mathematical expression of column variables from the program matrix.: `c` - the new constraint. **Throws:**: `java.lang.IllegalArgumentException` - if the variables in the provided expressions are not column variables of the program matrix.: `java.lang.NullPointerException` - if either argument is `null`.

---


### setRowConstraint

```
public final void setRowConstraint(java.lang.Object rowVariable,
                                   Interval c)
```

:   Changes the constraint of the provided row variable.

    :   **Parameters:**: `rowVariable` - the row variable in the program matrix whose constraint is to be changed.: `c` - the new constraint. **Throws:**: `java.lang.IllegalArgumentException` - if `rowVariable` is not a row variable of the program matrix.: `java.lang.NullPointerException` - if the provided constraint is `null`.

---


### setColumnConstraints

```
public final void setColumnConstraints(java.util.Map<?,Interval> columnConstraints)
```

:   Changes the constraints on more than one column variable.

    :   **Parameters:**: `columnConstraints` - column variables mapped to their respective constraints.

---


### setFluxConstraints

```
public final void setFluxConstraints(java.util.Map<java.lang.String,Interval> fluxConstraints)
```

:   Changes the flux constraints on more than one reaction.

    :   **Specified by:**: `setFluxConstraints` in interface `FluxCvComputer`
    :   **Parameters:**: `fluxConstraints` - reaction names mapped to their respective flux constraints.

---


### setRxnExprConstraints

```
public final void setRxnExprConstraints(java.util.Map<? extends MathExpr<java.lang.String>,Interval> exprConstraints)
```

:   Changes the constraints on more than one mathematical expression
    of fluxes.

    :   **Specified by:**: `setRxnExprConstraints` in interface `FluxCvComputer`
    :   **Parameters:**: `exprConstraints` - mathematical expressions of reaction names mapped to their new respective constraints.

---


### setColExprConstraints

```
public final void setColExprConstraints(java.util.Map<? extends MathExpr<?>,Interval> exprConstraints)
```

:   Changes the constraints on more than one mathematical expression
    of column variables.

    :   **Parameters:**: `exprConstraints` - mathematical expressions of column variables mapped to their new respective constraints.

---


### setRowConstraints

```
public final void setRowConstraints(java.util.Map<?,Interval> rowConstraints)
```

:   Changes the constraints on more than one row variable.

    :   **Parameters:**: `rowConstraints` - row variables mapped to their respective constraints.

---


### restoreColumnConstraint

```
public final void restoreColumnConstraint(java.lang.Object columnVariable)
```

:   Restores the constraint for the provided column variable
    to the value contained in the fluxome. If this variable is not
    present in the fluxome, its constraint is set to (-Infinity, Infinity).

    :   **Parameters:**: `columnVariable` - the column variable whose constraint is to be restored.

---


### restoreFluxConstraint

```
public final void restoreFluxConstraint(java.lang.String reactionName)
```

:   Restores the flux constraint for the reaction with the provided
    name to the value contained in the fluxome.

    :   **Specified by:**: `restoreFluxConstraint` in interface `FluxCvComputer`
    :   **Parameters:**: `reactionName` - the name of the reaction whose flux constraint is to be restored.

---


### restoreRxnExprConstraint

```
public final void restoreRxnExprConstraint(MathExpr<java.lang.String> rxnNameExpr)
```

:   Restores the constraint on the provided flux expression to the value
    contained in the fluxome.

    :   **Specified by:**: `restoreRxnExprConstraint` in interface `FluxCvComputer`
    :   **Parameters:**: `rxnNameExpr` - the expression of reaction names whose contraint is to be restored.

---


### restoreColExprConstraint

```
public final void restoreColExprConstraint(MathExpr<?> columnVarExpr)
```

:   Restores the constraint on the provided expression of column variables
    to the value contained in the fluxome. If this expression is not
    present in the fluxome, its constraint is set to (-Infinity, Infinity).

    :   **Parameters:**: `columnVarExpr` - the expression of column variables whose contraint is to be restored.

---


### restoreRowConstraint

```
public final void restoreRowConstraint(java.lang.Object rowVariable)
```

:   Restores the constraint on the provided row variable. If the provided
    variable is a chemical species, its constraint is set to [0, 0]. If the
    provided variable is not a chemical species, its constraint is set to
    (-Infinity, Infinity).

    :   **Parameters:**: `rowVariable` - a row variable from the program matrix.

---


### restoreColumnConstraints

```
public final void restoreColumnConstraints(java.util.Collection<?> columnVariables)
```

:   Restores the constraints for the provided column variables to those in
    the provided fluxome.

    :   **Parameters:**: `columnVariables` - a collection of column variables. **See Also:**: `restoreColumnConstraint(Object)`

---


### restoreFluxConstraints

```
public final void restoreFluxConstraints(java.util.Collection<java.lang.String> reactionNames)
```

:   Restores the constraints for the provided reaction names to those in the
    provided fluxome.

    :   **Specified by:**: `restoreFluxConstraints` in interface `FluxCvComputer`
    :   **Parameters:**: `reactionNames` - a collection of reaction names. **See Also:**: `restoreFluxConstraint(String)`

---


### restoreRxnExprConstraints

```
public final void restoreRxnExprConstraints(java.util.Collection<? extends MathExpr<java.lang.String>> rxnNameExprs)
```

:   Restores the constraints for the provided reaction name expressions to
    those in the provided fluxome.

    :   **Specified by:**: `restoreRxnExprConstraints` in interface `FluxCvComputer`
    :   **Parameters:**: `rxnNameExprs` - a collection of mathematical expressions of reaction names. **See Also:**: `restoreRxnExprConstraint(MathExpr)`

---


### restoreColExprConstraints

```
public final void restoreColExprConstraints(java.util.Collection<? extends MathExpr<?>> colVarExprs)
```

:   Restores the constraints for the provided column variable expressions to
    those in the provided fluxome.

    :   **Parameters:**: `colVarExprs` - a collection of mathematical expressions of column variables. **See Also:**: `restoreColExprConstraint(MathExpr)`

---


### restoreRowConstraints

```
public final void restoreRowConstraints(java.util.Collection<?> rowVariables)
```

:   Restores the constraints for the provided row variables to those in the
    provided fluxome.

    :   **Parameters:**: `rowVariables` - a collection of row variables. **See Also:**: `restoreRowConstraint(Object)`

---


### restoreColumnConstraints

```
public final void restoreColumnConstraints()
```

:   Restores the constraints on all column variables to those contained
    in the fluxome used by this optimization computer.

    :   **See Also:**: `restoreColumnConstraint(Object)`

---


### restoreFluxConstraints

```
public final void restoreFluxConstraints()
```

:   Restores the flux constraints for all reactions to those contained
    in the fluxome used by this optimization computer.

    :   **Specified by:**: `restoreFluxConstraints` in interface `FluxCvComputer`
    :   **See Also:**: `restoreFluxConstraint(String)`

---


### restoreRxnExprConstraints

```
public final void restoreRxnExprConstraints()
```

:   Restores the constraints on all mathematical expressions of reaction
    names.

    :   **Specified by:**: `restoreRxnExprConstraints` in interface `FluxCvComputer`
    :   **See Also:**: `restoreRxnExprConstraint(MathExpr)`

---


### restoreColExprConstraints

```
public final void restoreColExprConstraints()
```

:   Restores the constraints on all mathematical expressions of column
    variables.

    :   **See Also:**: `restoreColExprConstraint(MathExpr)`

---


### restoreRowConstraints

```
public final void restoreRowConstraints()
```

:   Restores the constraints on all row variables to those contained in
    the fluxome used by this optimization computer.

    :   **See Also:**: `restoreRowConstraint(Object)`

---


### getColumnVariables

```
public java.util.Set<?> getColumnVariables()
```

:   Returns the set of all column variables in the program matrix.

    :   **Returns:**: the set of all column variables in the program matrix.

---


### getRxnExprs

```
public java.util.Set<MathExpr<java.lang.String>> getRxnExprs()
```

:   Returns the set of all mathematical expressions of reaction names.

    :   **Returns:**: the set of all mathematical expressions of reaction names.

---


### getColExprs

```
public java.util.Set<MathExpr<?>> getColExprs()
```

:   Returns the set of all mathematical expressions of column variables.

    :   **Returns:**: the set of all mathematical expressions of column variables.

---


### getRowVariables

```
public java.util.Set<?> getRowVariables()
```

:   Returns the set of all row variables in the program matrix.

    :   **Returns:**: the set of all row variables in the program matrix.

---


### getColumnConstraint

```
public Interval getColumnConstraint(java.lang.Object columnVariable)
```

:   Returns the current constraint on the provided column variable.

    :   **Parameters:**: `columnVariable` - the column variable whose constraint is to be returned. **Returns:**: the current constraint on the provided column variable.

---


### getFluxConstraint

```
public Interval getFluxConstraint(java.lang.String reactionName)
```

:   Returns the current flux constraint on the reaction with the provided
    name.

    :   **Specified by:**: `getFluxConstraint` in interface `FluxCvComputer`
    :   **Parameters:**: `reactionName` - the name of the reaction whose flux constraint is to be returned. **Returns:**: the current flux constraint on the reaction with the provided name.

---


### getRxnExprConstraint

```
public Interval getRxnExprConstraint(MathExpr<java.lang.String> rxnNameExpr)
```

:   Returns the current constraint on the provided expression.

    :   **Specified by:**: `getRxnExprConstraint` in interface `FluxCvComputer`
    :   **Parameters:**: `rxnNameExpr` - the mathematical expression of reaction names whose constraint is to be returned. **Returns:**: the current constraint on the provided expression.

---


### getColExprConstraint

```
public Interval getColExprConstraint(MathExpr<?> columnVariableExpr)
```

:   Returns the current constraint on the provided expression.

    :   **Parameters:**: `columnVariableExpr` - the mathematical expression of column variables whose constraint is to be returned. **Returns:**: the current constraint on the provided expression.

---


### getRowConstraint

```
public Interval getRowConstraint(java.lang.Object rowVariable)
```

:   Returns the current constraint on the provided row variable.

    :   **Parameters:**: `rowVariable` - the row variable whose constraint is to be returned. **Returns:**: the current constraint on the provided row variable.

---


### getColumnConstraints

```
public java.util.Map<?,Interval> getColumnConstraints()
```

:   Returns the current constraints for each column variable in
    the program matrix.

    :   **Returns:**: each column variable in the program matrix mapped to its current constraint.

---


### getFluxConstraints

```
public java.util.Map<java.lang.String,Interval> getFluxConstraints()
```

:   Returns all current flux constraints for each reaction
    in the fluxome used by this optimization computer.

    :   **Specified by:**: `getFluxConstraints` in interface `FluxCvComputer`
    :   **Returns:**: the names of all reactions mapped to their current flux constraint.

---


### getColExprConstraints

```
public java.util.Map<MathExpr<?>,Interval> getColExprConstraints()
```

:   Returns all current constraints on mathematical expressions
    of column variables.

    :   **Returns:**: all mathematical expressions of column variables contained in this computer mapped to their current constaint.

---


### getRxnExprConstraints

```
public java.util.Map<MathExpr<java.lang.String>,Interval> getRxnExprConstraints()
```

:   Returns all current constraints on mathematical expressions
    of reaction names.

    :   **Specified by:**: `getRxnExprConstraints` in interface `FluxCvComputer`
    :   **Returns:**: all mathematical expressions of reaction names contained in this computer mapped to their current constraint.

---


### getRowConstraints

```
public java.util.Map<?,Interval> getRowConstraints()
```

:   Returns all the current constraints on row variables.

    :   **Returns:**: all row variables mapped to their current constraint.

---


### getOptimal

```
public final FbaOptComputer.Results getOptimal(java.lang.String reactionName,
                                               OptimizationSense sense)
```

:   Returns the results of computing the optimal flux of the reaction
    with the provided name.

    :   **Parameters:**: `reactionName` - the name of the reaction whose flux is to be optimized.: `sense` - the sense, or direction, of optimization. **Returns:**: the results of computing the optimal flux of the reaction with the provided name.

---


### getOptimal

```
public final FbaOptComputer.Results getOptimal(MathExpr<?> objective,
                                               OptimizationSense sense)
```

:   Returns the results of computing the optimal value of the
    provided objective function.

    :   **Parameters:**: `objective` - a mathematical expression of column variables.: `sense` - the sense, or direction, of optimization. **Returns:**: the results of computing the optimal value of the provided objective function.

---


### getMax

```
public final FbaOptComputer.Results getMax(MathExpr<?> objective)
```

:   Returns the results of computing the maximum value of the
    provided objective function.

    :   **Parameters:**: `objective` - a mathematical expression of column variables. **Returns:**: the results of computing the maximum value of the provided objective function.

---


### getMin

```
public final FbaOptComputer.Results getMin(MathExpr<?> objective)
```

:   Returns the results of computing the minimum value of the
    provided objective function.

    :   **Parameters:**: `objective` - a mathematical expression of column variables. **Returns:**: the results of computing the minimum value of the provided objective function.

---


### getMaximum

```
public double getMaximum(MathExpr<?> objective)
```

:   Computes the maximum value of the provided objective function.

    :   **Parameters:**: `objective` - a mathematical expression whose variables are column variables in the program matrix. **Returns:**: the maximum value the provided mathematical expression can assume. **Throws:**: `java.lang.NullPointerException` - if the provided expression is null.: `FbaOptException` - if an optimal solution cannot be found.: `java.lang.IllegalArgumentException` - if the provided expression contains a variable that is not a column variable in the program matrix.

---


### getFluxMax

```
public double getFluxMax(java.lang.String reactionName)
```

:   Computes the maximum flux of the reaction with the provided name.

    :   **Parameters:**: `reactionName` - the name of the reaction whose maximum flux will be computed. **Returns:**: the maximum flux of the reaction with the provided name. **Throws:**: `java.lang.IllegalArgumentException` - if the provided string is not a reaction name of the fluxome used by this optimization computer.

---


### getColumnMax

```
public double getColumnMax(java.lang.Object columnVariable)
```

:   Computes the maximum value of the provided column variable.

    :   **Parameters:**: `columnVariable` - a column variable from the program matrix whose maximum value is to be computed. **Returns:**: the maximum value the provided column variable can assume. **Throws:**: `java.lang.IllegalArgumentException` - if the provided object is not a column variable in the program matrix.

---


### getMaxima

```
public <V,E extends MathExpr<V>> java.util.Map<E,java.lang.Double> getMaxima(java.util.Collection<E> objectives)
```

:   Computes the maximum value of each provided objective function.

    :   **Type Parameters:**: `V` - the variable type.: `E` - the mathematical expression type. **Parameters:**: `objectives` - a collection of mathematical expressions whose variables are column variables in the program matrix. **Returns:**: the provided mathematical expressions mapped to their computed maximum value. **Throws:**: `java.lang.NullPointerException` - if the provided collection, or any of its elements, is `null`.: `java.lang.IllegalArgumentException` - if one of the provided mathematical expressions contains a variable that is not a column variable.

---


### getFluxMaxima

```
public java.util.Map<java.lang.String,java.lang.Double> getFluxMaxima(java.util.Collection<java.lang.String> reactionNames)
```

:   Computes the maximum flux of each reaction with the provided names.

    :   **Parameters:**: `reactionNames` - a collection of reaction names from the fluxome used by this optimization computer. **Returns:**: the provided reaction names mapped to their computed maximum flux. **Throws:**: `java.lang.NullPointerException` - if the provided collection is `null`.: `java.lang.IllegalArgumentException` - if an element in the provided collection is not a reaction name.

---


### getColumnMaxima

```
public <T> java.util.Map<T,java.lang.Double> getColumnMaxima(java.util.Collection<T> columnVariables)
```

:   Computes the maximum values of each provided column variable.

    :   **Type Parameters:**: `T` - the column variable type. **Parameters:**: `columnVariables` - a collection of column variables in the program matrix. **Returns:**: the provided column variables mapped to their computed maximum value. **Throws:**: `java.lang.IllegalArgumentException` - if an element in the provided collection is not a legitimate column variable.

---


### getMinimum

```
public double getMinimum(MathExpr<?> objective)
```

:   Computes the minimum value of the provided objective function.

    :   **Parameters:**: `objective` - a mathematical expression whose variables are column variables in the program matrix. **Returns:**: the minimum value of the provided objective function. **Throws:**: `java.lang.IllegalArgumentException` - if a variable in the provided expression is not a column variable in the program matrix.

---


### getFluxMin

```
public double getFluxMin(java.lang.String reactionName)
```

:   Computes the minimum flux of the reaction with the provided name.

    :   **Parameters:**: `reactionName` - a reaction name from the fluxome used by this optimization computer. **Returns:**: the minimum flux of the reaction with the provided name. **Throws:**: `java.lang.IllegalArgumentException` - if the provided string is not a reaction name.

---


### getColumnMin

```
public double getColumnMin(java.lang.Object columnVariable)
```

:   Computes the minimum value of the provided column variable.

    :   **Parameters:**: `columnVariable` - a column variable from the program matrix. **Returns:**: the computed minimum value of the provided column variable. **Throws:**: `java.lang.IllegalArgumentException` - if the provided object is not a column variable in the program matrix.

---


### getMinima

```
public <V,E extends MathExpr<V>> java.util.Map<E,java.lang.Double> getMinima(java.util.Collection<E> objectives)
```

:   Computes the minimum value of each provided objective function.

    :   **Type Parameters:**: `V` - the variable type.: `E` - the mathematical expression type. **Parameters:**: `objectives` - a collection of mathematical expressions whose variables are column variables in the program matrix. **Returns:**: the provided mathematical expressions mapped to their computed minimum value. **Throws:**: `java.lang.NullPointerException` - if the provided collection, or any of its elements, is `null`.: `java.lang.IllegalArgumentException` - if one of the provided mathematical expressions contains a variable that is not a column variable.

---


### getFluxMinima

```
public java.util.Map<java.lang.String,java.lang.Double> getFluxMinima(java.util.Collection<java.lang.String> reactionNames)
```

:   Computes the minimum flux of each reaction with the provided names.

    :   **Parameters:**: `reactionNames` - a collection of reaction names from the fluxome used by this optimization computer. **Returns:**: the provided reaction names mapped to their computed minimum flux. **Throws:**: `java.lang.NullPointerException` - if the provided collection is `null`.: `java.lang.IllegalArgumentException` - if an element in the provided collection is not a reaction name.

---


### getColumnMinima

```
public <T> java.util.Map<T,java.lang.Double> getColumnMinima(java.util.Collection<T> columnVariables)
```

:   Computes the minimum values of each provided column variable.

    :   **Type Parameters:**: `T` - the column variable type. **Parameters:**: `columnVariables` - a collection of column variables in the program matrix. **Returns:**: the provided column variables mapped to their computed minimum value. **Throws:**: `java.lang.IllegalArgumentException` - if an element in the provided collection is not a legitimate column variable.

---


### checkRowVariable

```
protected void checkRowVariable(java.lang.Object obj)
```

:   Ensures the provided object is row variable.

    :   **Parameters:**: `obj` - the object to check. **Throws:**: `java.lang.IllegalArgumentException` - if the provided object is not a row variable in the program matrix.

---


### checkColumnVariable

```
protected void checkColumnVariable(java.lang.Object obj)
```

:   Ensures the provided object is column variable.

    :   **Parameters:**: `obj` - the object to check. **Throws:**: `java.lang.IllegalArgumentException` - if the provided object is not a column variable in the program matrix.

---


### checkColumnVariables

```
protected void checkColumnVariables(java.util.Collection<?> c)
```

:   Ensures each element of the provided collection is column variable.

    :   **Parameters:**: `c` - the collection of objects to check. **Throws:**: `java.lang.IllegalArgumentException` - if an element in the provided collection is not a column variable in the program matrix.

---


### checkColExpr

```
protected void checkColExpr(MathExpr<?> e)
```

:   Ensures all the variables in the provided expression are valid column
    variables.

    :   **Parameters:**: `e` - the expression to check. **Throws:**: `java.lang.IllegalArgumentException` - if a variable in the provided expression is not a column variable in the program matrix.

---


### checkRxnName

```
protected void checkRxnName(java.lang.String string)
```

:   Ensures the provided string is the name of a reaction in the fluxome
    used by this optimization computer.

    :   **Parameters:**: `string` - the string to check. **Throws:**: `java.lang.IllegalArgumentException` - if the provided string is not the name of a reaction in the fluxome used by this optimization computer.

---


### checkRxnNames

```
protected void checkRxnNames(java.util.Collection<java.lang.String> c)
```

:   Ensures each element of the provided collection is reaction name.

    :   **Parameters:**: `c` - the collection of strings to check. **Throws:**: `java.lang.IllegalArgumentException` - if a string in the provided collection is not the name of a reaction in the fluxome used by this optimization computer.

---


### checkRxnExpr

```
protected void checkRxnExpr(MathExpr<java.lang.String> e)
```

:   Ensures all the variables in the provided expression are reaction names.

    :   **Parameters:**: `e` - the expression to check. **Throws:**: `java.lang.IllegalArgumentException` - if a variable in the provided expression is not the name of a reaction in the fluxome used by this optimization computer.

---


### checkNumber

```
protected void checkNumber(double number)
```

:   Ensures the provided number is finite and not NaN.

    :   **Parameters:**: `number` - the number to check. **Throws:**: `java.lang.IllegalArgumentException` - if the provided number is infinite or NaN.

---


### checkChemSpecies

```
protected void checkChemSpecies(java.lang.String string)
```

:   Ensures the provided string is the name of a chemical species in the fluxome
    used by this optimization computer.

    :   **Parameters:**: `string` - the string to check. **Throws:**: `java.lang.IllegalArgumentException` - if the provided string is not the name of a chemical species in the fluxome used by this optimization computer.

---


### checkRxnExprConstraint

```
protected void checkRxnExprConstraint(Interval c)
```

:   Ensures the provided constraint is valid for reaction name-expressions
    for the fluxome used by this optimization computer.

    :   **Parameters:**: `c` - the constraint to check. **Throws:**: `java.lang.NullPointerException` - if the provided constraint is `null`.

---


### checkFluxConstraint

```
protected void checkFluxConstraint(Interval c,
                                   java.lang.String rxnName,
                                   double tolerance)
```

:   Ensures the provided constraint is a valid flux constraint
    in the fluxome used by this optimization computer.

    :   **Parameters:**: `c` - the constraint to check.: `rxnName` - the name of the reaction for which this constraint applies.: `tolerance` - the allowable amount by which the bounds of the provided constraint can violate the maximum bounds defined the fluxome used by this optimization computer. **Throws:**: `java.lang.IllegalArgumentException` - if the provided constraint is not a valid flux constraint.

---


### getExpr

```
protected <V> LinearComb<V> getExpr(V columnVariable)
```

:   Converts a column variable to a single-term linear combination.

    :   **Type Parameters:**: `V` - the column variable type. **Parameters:**: `columnVariable` - a column variable from the program matrix. **Returns:**: a single-term linear combination containing the provided column variable. The coefficient of this term equals 1, and the constant equals 0. **Throws:**: `java.lang.IllegalArgumentException` - if the provided object is not a column variable in the program matrix.

---


### getRxnExpr

```
protected LinearComb<java.lang.String> getRxnExpr(java.lang.String reactionName)
```

:   Converts a reaction name to a single-term linear combination.

    :   **Parameters:**: `reactionName` - a reaction name from the fluxome used to create this optimization computer. **Returns:**: a single-term linear combination containing the provided reaction name. The coefficient of this term equals 1, and the constant equals 0. **Throws:**: `java.lang.IllegalArgumentException` - if the provided string is not a reaction name in the fluxome used by this optimization computer.

---


### getRxnExpr

```
protected LinearComb<java.lang.String> getRxnExpr(java.util.Collection<java.lang.String> rxnNames)
```

:   Converts a collection of reaction names to a linear combination.

    :   **Parameters:**: `rxnNames` - a collection of reaction names from the fluxome used by this optimization computer. **Returns:**: a linear combination containing the provided reaction names. The coefficient of each term in the expression equals 1, and the constant equals 0. **Throws:**: `java.lang.IllegalArgumentException` - if an element in the provided collection is not a reaction name in the fluxome used by this optimization computer.

---


### getExpr

```
protected <V> LinearComb<V> getExpr(java.util.Collection<V> columnVariables)
```

:   Converts a collection of column variables to a linear combination.

    :   **Type Parameters:**: `V` - the column variable type. **Parameters:**: `columnVariables` - a collection of column variables contained in the program matrix. **Returns:**: a linear combination containing the provided column variables. The coefficient of each term in the expression equals 1, and the constant equals 0. **Throws:**: `java.lang.IllegalArgumentException` - if an element in the provided collection is not a column variable in the program matrix.

---


### checkSolutionVector

```
protected void checkSolutionVector(java.util.Map<?,java.lang.Double> solutionVector)
```

:   Ensures the provided solution vector does not violate the defined
    constraints of the system.

    :   **Parameters:**: `solutionVector` - column variables mapped to their respective numerical values. **Throws:**: `ConstraintViolationException` - if a value in the provided vector violates its defined constraints by more than the defined constraint tolerance.: `java.lang.IllegalArgumentException` - if a key in the provided map is not a legitimate column variable in the program matrix.: `java.lang.NullPointerException` - if a value in the provided map is `null`.

---


### checkColumnValue

```
protected void checkColumnValue(java.lang.Object columnVariable,
                                java.lang.Double value)
```

:   Ensures the provided numerical value does not violate the defined
    constraint of the provided column variable.

    :   **Parameters:**: `columnVariable` - the column variable whose value and constraint will be compared.: `value` - the numerical value of the provided column variable. **Throws:**: `ConstraintViolationException` - if the provided value violates the defined constraint for the provided column variable by more than the defined constraint tolerance.: `java.lang.IllegalArgumentException` - if the provided column variable does not exist in the program matrix.: `java.lang.NullPointerException` - if the provided value is `null`.

---


### checkColumnValue

```
protected void checkColumnValue(java.lang.Object columnVariable,
                                double value)
```

:   Ensures the provided numerical value does not violate the defined
    constraint of the provided column variable.

    :   **Parameters:**: `columnVariable` - the column variable whose value and constraint will be compared.: `value` - the numerical value of the provided column variable. **Throws:**: `ConstraintViolationException` - if the provided value violates the defined constraint for the provided column variable by more than the defined constraint tolerance.: `java.lang.IllegalArgumentException` - if the provided column variable does not exist in the program matrix.


---


|  |  |  |
| --- | --- | --- |
| **Overview** \| **Package** \| **Class** \| **Use** \| **Tree** \| **Deprecated** \| **Index** \| **Help** | |  |
| **PREV CLASS**   **NEXT CLASS** | **FRAMES**    **NO FRAMES**     **All Classes** |  |
| SUMMARY: NESTED \| FIELD \| CONSTR \| METHOD | DETAIL: FIELD \| CONSTR \| METHOD |  |


---
